# Risk for hypoglycemic emergency with levofloxacin use, a population-based propensity score matched nested case-control study

**DOI:** 10.1371/journal.pone.0266471

**Published:** 2022-04-04

**Authors:** Shu-Hui Liao, Sung-Yuan Hu, Chorng-Kuang How, Vivian Chia-Rong Hsieh, Chia-Ming Chan, Chien-Shan Chiu, Ming-Shun Hsieh

**Affiliations:** 1 Department of Pathology and Laboratory, Taipei Veterans General Hospital, Taoyuan Branch, Taoyuan, Taiwan; 2 Department of Emergency Medicine, Taichung Veterans General Hospital, Taichung, Taiwan; 3 Chung Shan Medical University, Taichung, Taiwan; 4 Department of Emergency Medicine, Taipei Veterans General Hospital, Taipei, Taiwan; 5 School of Medicine, National Yang Ming Chiao Tung University, Taipei, Taiwan; 6 Kinmen Hospital, Ministry of Health and Welfare, Kinmen, Taiwan; 7 Department of Health Services Administration, China Medical University, Taichung, Taiwan; 8 Department of Dermatology, Taichung Veterans General Hospital, Taichung, Taiwan; 9 Institute of Biomedical Sciences, National Chung Hsing University, Taichung, Taiwan; 10 Department of Emergency Medicine, Taipei Veterans General Hospital, Taoyuan Branch, Taoyuan, Taiwan; Hualien Tzu Chi Hospital Buddhist Tzu Chi Medical Foundation, TAIWAN

## Abstract

Potential association between oral levofloxacin use and hypoglycemic emergency (HE) have been established. However, a large epidemiological study is required to verify this observation. This study aimed to determine if use of oral levofloxacin increased the risk of HE. The nationwide database between 1999 and 2013, including 1.6 million patients with type 2 diabetes (T2D), was used to conduct a nested case-control study. Cases and controls comprised of patients with and without HE, respectively. To avoid indication bias the control subjects were chosen through propensity score matching with cases in a 10-fold ratio. T2D severity was classified based on the adjusted diabetic complication severity index score. 26,695 and 266,950 matched patients with T2D, were finally used as cases and controls, respectively, for the analysis. Multivariate logistic regression analysis showed that antibiotic use was associated with an increased risk for HE (adjusted odds ratio (aOR) = 6.08, 95% confidence interval (95% CI): 5.79–6.38). When compared with antibiotic non-users, those who used fluoroquinolones and sulfonamides displayed the highest (aOR = 12.05, 95% CI: 10.66–13.61) and second highest (aOR = 7.20, 95% CI: 6.29–8.24) risks of HE, respectively. The associated risk for HE was significantly higher with levofloxacin than that with cephalosporins (aOR = 5.13, 95% CI: 2.28–11.52) and penicillin (aOR = 9.40, 95% CI: 2.25–39.24). In the joint effect analyses, the risk for HE increased with the combination of levofloxacin with insulin (aOR = 8.42, 95% CI: 1.91–37.00) or sulfonylurea (aOR = 3.56, 95% CI: 1.12–11.33). Use of oral levofloxacin, compared to that of other antibiotics, was found to be significantly associated with HE in T2D patients. Clinicians should exercise caution while prescribing levofloxacin, especially when combined with insulin or sulfonylurea.

## Introduction

Patients with type 2 diabetic mellitus (T2D) are susceptible to secondary infections that can further disrupt their blood sugar balance and therefore, induce hypoglycemia or hyperglycemia. For hypoglycemia, the risk factors include inappropriate dosing of antidiabetic drugs, dietary indiscretion, and adverse drug side effects [1–4]. Hypoglycemia have been often reported in connection with impaired renal function and/or among older patients receiving oral antidiabetic drugs, especially sulfonylureas or insulin [5]. Recurrent hypoglycemia can cause severe brain injury and long-term neurological complications [6,7].

Fluoroquinolones belongs to a class of antibiotics that inhibits bacterial DNA synthesis, and are one of the most commonly prescribed antibiotics, worldwide, primarily for the treatment of respiratory and urinary tract infections [8]. The development of latest generation of fluoroquinolones, extended the coverage of bacteria from the traditional spectrum to anaerobes. However, the unregulated use of fluoroquinolones can lead to an increasing emergence of bacterial drug resistance. Levofloxacin is currently one of the most commonly used oral antibiotics. However, several case series have reported the association between levofloxacin use and hypoglycemia emergency (HE) [9,10]. In 2018, the FDA announced again a safety warning on the risk for hypoglycemia with levofloxacin use [11]. However, the exact nature of association between levofloxacin use and the risk of hypoglycemia have not been definitely established, until now, due to the limited number of cases (mostly from case reports). Therefore, in this study, we conducted a nested case-control analysis on a specially applied nationwide diabetes database from Taiwan between 1999 and 2013, to resolve an old but important dilemma on the association between levofloxacin use and HE, and provided comprehensive proof that compared with other antibiotics, it is significantly associated with the risk for HE in patients with T2D.

## Methods

### Data sources

1.6 million patients of Taiwanese origin, from a specially applied diabetes database from the Taiwan National Health Insurance Research Database (NHIRD) were included in this study. NHIRD was launched by the National Health Insurance Administration (NHIA) of Taiwan in 1995 and currently provides nearly an overall coverage of more than 23.03 million residents (>99% of the entire population). The NHIA releases de-identified patient information and claims data to the National Health Research Institute, which compiles it into NHIRD. The data is of high quality which has been confirmed by several prior studies [12–15] and the confidentiality and credibility of the data is strictly maintained in accordance with the NHIRD regulations.

### Disease definitions

The diagnosis codes, in accordance with the International Classification of Diseases, 9^th^ Revision, Clinical Modification (ICD-9-CM), is used throughout this study. The diagnostic accuracy of T2D in the NHIRD have been validated in a prior study [16]. Baseline comorbidities such as chronic kidney disease (CKD), chronic obstructive pulmonary disease (COPD), hypertension, hyperlipidemia, congestive heart failure, liver cirrhosis. cancer, ischemic stroke, hemorrhagic stroke, seizure, dementia, Parkinson’s disease, were defined using the ICD-9-CM. For example, if a patient was defined to have a baseline comorbidity, such as COPD, it was ensured that they had at least one of the following; (1) ≥2 outpatient visits for the same main diagnosis or (2) one specific hospitalization diagnosis record for the baseline comorbidity such as COPD. Due to privacy of individual identity, information about individuals’ income could not be directly obtained; instead, the insurance premium fees, were widely adopted and recorded, as a surrogate for household income level, in this administrative database [17].

The date of initial diagnosis of hypoglycemia in the emergency department (ED) were recorded as the index date between the study period 1999 and 2013. Any other diagnoses, made within one year prior to the index date, were considered as the underlying comorbidities of a patient. The patients were classified based on their usage of certain drugs or usual medications for chronic diseases, for at least one week within a three-month period. The usual medications included biguanides, DDP-4 inhibitors, sulfonylureas, thiazolidinediones, insulin, nonsteroidal anti-inflammatory drugs, aspirin, and statins. Even though patients with type 1 diabetes were not included in the study, certain type 2 diabetic patients with poor blood sugar control were prescribed a combined use of insulin (such as basal insulin at night) and oral medications concurrently.

### T2D severity

The severity of T2D plays an important role in the occurrence of HE. The adjusted Diabetes Complications Severity Index score (aDCSI score) was used as a reference measure for the severity of T2D [18,19]. The patients in the case and control groups were required to have been diagnosed as T2D for at least one year prior to the index date to allow evaluation of diabetic complication burdens. The aDCSI score is a useful tool to adjust for the baseline severity of diabetic complications. It predicts hospital outcomes, and have been validated in the NHIRD [20]. It includes the following seven categories of diabetic complications: cardiovascular disease, nephropathy, neuropathy, stroke, peripheral vascular disease, retinopathy, and metabolic emergency events.

### Case and control group selection

A nested case-control study was conducted. Both the case and the control subjects comprised of only patients with T2D. Patients aged <20 or >90 years and patients with type-I diabetes were excluded from the study. First, the case subjects were chosen based on the following criteria; (i) having a HE necessitating ED visit during the study period with a record of receiving high concentration (10% or 50%) of dextrose infusion, and (ii) intake of certain oral antibiotics within one week prior to the HE [21]. Only the first episode of HE was considered for inclusion into further study. All cases with repeated ER visits were excluded from further analysis on the grounds of poor self-care or drug compliance. Taiwan government has strict regulations regarding antibiotics that could only be obtained through prescription from registered physicians. Usage of all prescribed antibiotics were recorded, tabulated, and calculated.

**Fig 1:**
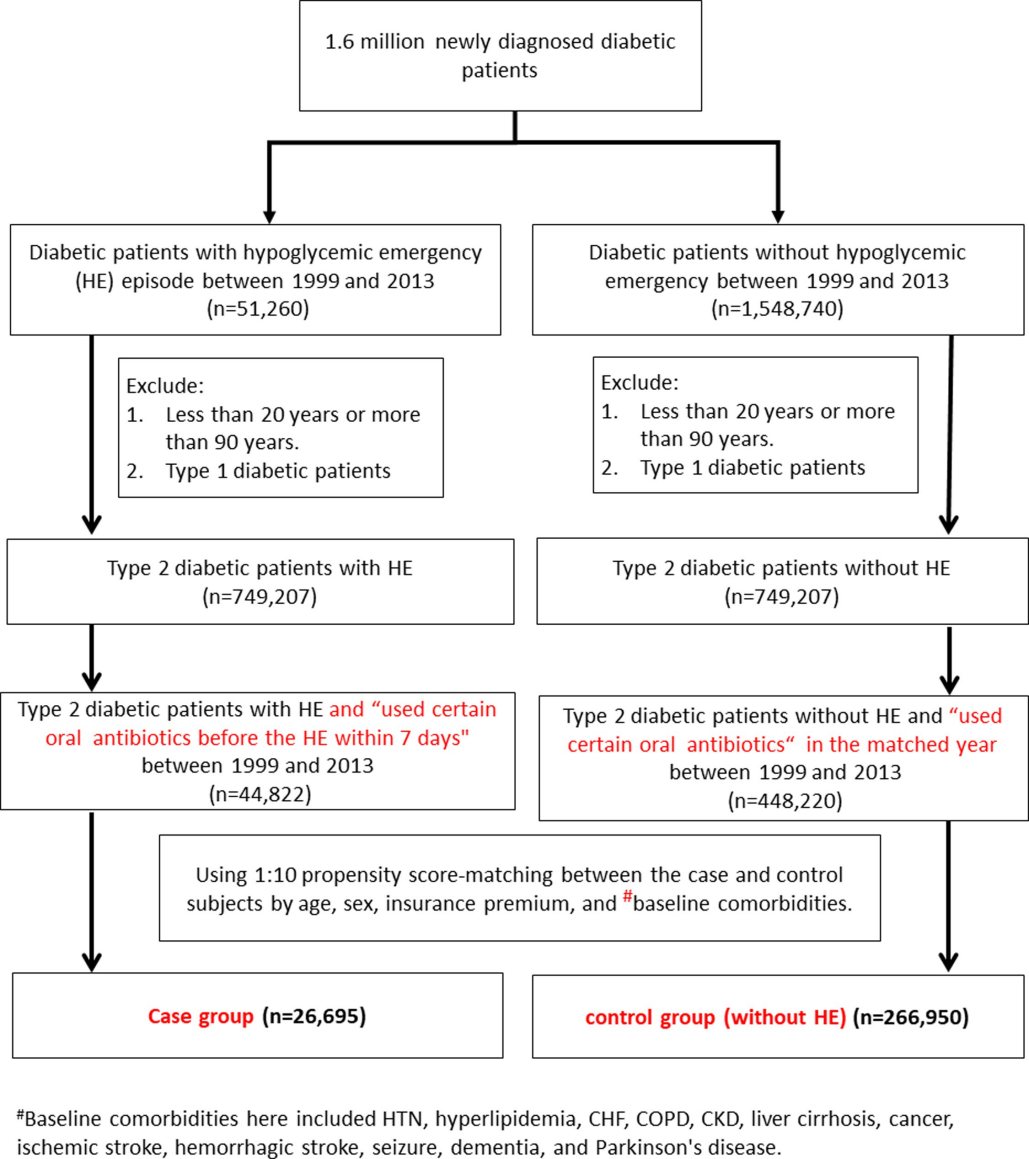
Selection algorithm for the case and control subjects in the study design.

The control subjects were defined as those who did not have HE but took oral antibiotics during a <7 days duration within the follow up period. The control subjects were retrieved by the PS method of "nearest neighbor matching". The first day of receiving prescriptions of oral antibiotics by the control subjects was defined as the index date. This was also matched to the calendar year for the case subjects. it was ensured that there was no HE in the control group one week prior to the index date. The algorithm of participant selection is displayed in Fig 1.

As the NHIRD contains de-identified secondary data for research, this study was exempted from the requirement for informed consent from participants. This study was approved by the institutional review board of China Medical University (IRB# CMUH104-REC2-115).

### Propensity score (PS) matching

Developing and applying the propensity score (PS) matching in an observational study to balance the probability of exposure to a specific treatment, based on the observed variables, remained popular and practical [22,23]. The patients were "pseudo-randomized" (or "quasi-randomized") according to the scores, calculated from their demographic and baseline factors [24].

The most challenging aspect of this study was the elimination of drug selection bias to the infected patients whose antibiotic treatments were arbitrary, if within the guideline. Some patients may have had different infections but received the same antibiotics, whereas others may have had the same infections but received different kinds of antibiotics. And these were at the physician’s discretion at that time; therefore, many confounding factors were not controllable.

In this study, PS matching was accordingly chosen and conducted to address the primary and inevitable indication bias. After the PS matching, the chance of receiving antibiotics would become nearly balanced between the case and control subjects [25].

In the PS matching, the scores indicated the probability that certain kind of antibiotic would be prescribed to the patients with infection. The PS was calculated by using multivariate logistic regression to adjust for the observed and available covariates. The PS matching was employed in a 1:10 ratio to match the case and control subjects. The scores were calculated by the logistic regression, including age, sex, household income, and baseline comorbidities (not including the DM severity (aDCSI score) and individual medications). One case was matched with 10 controls, according to the "nearest neighbor matching" (also called "greedy matching") [26,27]. Thus, the study dataset was constructed that comprised of PS matched case and control subjects. A standardized mean difference ≤0.05 between the study and control subjects indicated a negligible difference between the matched cases and controls, for age, sex, household income, and baseline comorbidities, as shown in Table 1. The aDCSI score and individual medications were not matched using PS between the study cases and control subjects.

**Table 1:** Demographic and clinical characteristics in patients with type 2 diabetes with and without hypoglycemic emergency after propensity score-matching.

Variables	Hypoglycemia emergency				Standardized mean difference^#^
	Yes (n = 26,695)		No (n = 266,950)		
	n	%	n	%	
**Sex**					
Female	13240	49.60	134480	50.38	0.016
Male	13455	50.40	132470	49.62	0.016
**Age group, years**					
20–39	1387	5.2	12121	4.54	0.030
40–59	7388	27.68	73541	27.55	0.003
60–79	14560	54.54	149787	56.11	0.032
≥80 years	3360	12.59	31501	11.8	0.024
Mean (SD)	65.41 (13.73)		64.71 (13.02)		0.053
**Insurance premium (NT dollars)**					
<20000	13011	48.74	130876	49.03	0.006
≥20000 to <40000	10989	41.17	107423	40.24	0.019
≥40000 to <60000	2038	7.63	21826	8.18	0.020
≥60000	657	2.46	6825	2.56	0.006
**Baseline comorbidity**					
Hypertension	19875	74.45	200208	75.00	0.013
Hyperlipidemia	16223	60.77	163133	61.11	0.007
Congestive heart failure	3810	14.27	37475	14.04	0.007
Chronic obstructive pulmonary disease	9841	36.86	96985	36.33	0.011
Chronic kidney disease	8308	31.12	84717	31.74	0.013
Liver cirrhosis	1737	6.51	15518	5.81	0.029
Cancer	3756	14.07	37228	13.95	0.004
Ischemic stroke	4719	17.68	49052	18.37	0.018
Hemorrhagic stroke	905	3.39	8476	3.18	0.012
Seizure	733	2.75	6066	2.27	0.030
Dementia	2245	8.41	20584	7.71	0.026
Parkinson’s disease	1126	4.22	9721	3.64	0.030
aDCSI score*					
0	8220	30.79	141939	53.17	0.466
1	4772	17.88	55505	20.79	0.074
2	6211	23.27	43198	16.18	0.179
3	3121	11.69	14431	5.41	0.226
4	2432	9.11	8096	3.03	0.257
≥5	1939	7.26	3781	1.42	0.290
Medications*					
Biguanides	15747	58.99	89409	33.49	0.529
DDP-4 inhibitors	2289	8.57	10668	4	0.189
Sulfonylureas	18491	69.27	90281	33.82	0.759
TZD	3725	13.95	14246	5.34	0.295
Other oral antidiabetic drugs	5377	20.14	22128	8.29	0.344
Insulin	6277	23.51	9504	3.56	0.610
NSAIDs	10786	40.4	90258	33.81	0.137
Aspirin	2489	9.32	20665	7.74	0.057
Statins	7517	28.16	59977	22.47	0.131

aDCSI score, adjusted diabetic complication severity index score; DPP-4, dipeptidyl peptidase-4; NSAID, non-steroidal anti-inflammatory drug; NT dollars, national Taiwan dollars; SD, standard deviation; TZD, thiazolidinedione; ^**#**^The PS matching included age, sex, insurance premium, and baseline.

*The aDCSI score, and medications were not included in the PS matching.

### Statistical analyses

Differences in demographic and clinical characteristics and baseline comorbidities were examined using the chi-square test and two-sample t-test. Odds ratio (OR) with 95% confidence interval (95% CI) was calculated for each variable in the logistic regression model. Adjusted ORs for HE were obtained by multivariate logistic regression analysis. The covariates included were different from the ones used for PS matching; DM severity (aDCSI score) and individual medications were added into the regression model for adjustment.

Comparison of HE events between antibiotic users and non-users were conducted. The antibiotics included fluoroquinolones, cephalosporins, penicillin, macrolides, sulfonamide, tetracycline, and metronidazole. Fluoroquinolones were further compared with cephalosporins and penicillin for the risk of HE. Finally, a joint effect analysis was performed on the combined use of levofloxacin and antidiabetic drugs.

All statistical analyses were performed using SAS 9.4 statistical package (SAS Institute Inc., Cary, NC, USA). A P-value of 0.05 was set as the threshold of significance.

## Results

**Fig 2:**
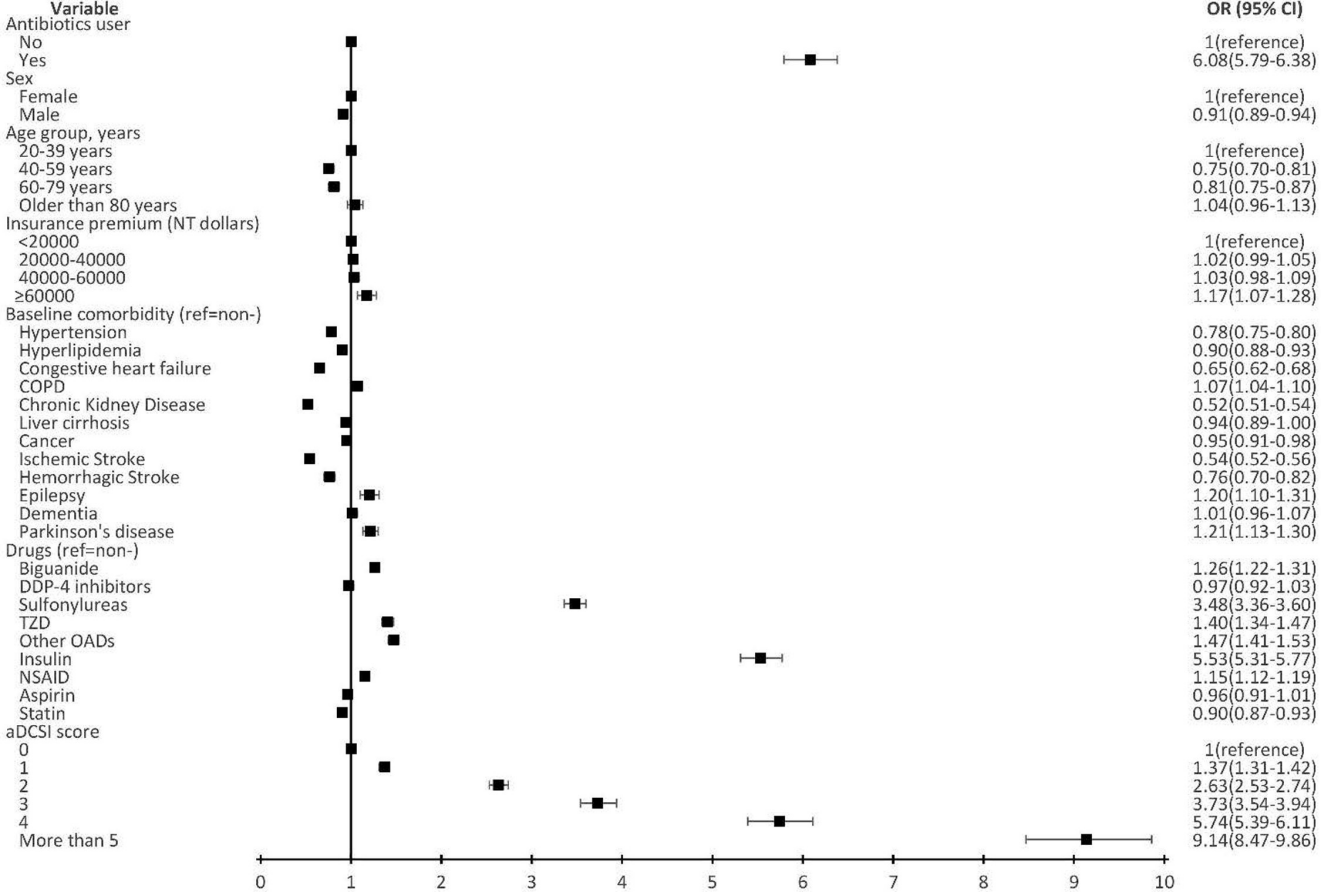
Logistic regression model to evaluate the risk for hypoglycemic emergency. OR: Odds ratio, CI: 95% Confidence Interval. The black boxes denote the ORs.

26,695 and 266,950 PS-matched patients, all with T2D, were enrolled as cases and controls, respectively. Comparisons of the demographic characteristics, and clinical characteristics, individual medications, and aDCSI scores between the two groups are presented in **Table 1**. Using the multivariate logistic regression model, antibiotic use, compared with no antibiotic use, was found to be associated with an increased risk for HE (adjusted odds ratio (aOR) = 6.08, 95% confidence interval (95% CI): 5.79–6.38) (**Fig 2**).

As opposed to antibiotic non-users, those who used fluoroquinolones and sulfonamides had the highest (aOR = 12.05, 95% CI: 10.66–13.61) and second highest (aOR = 7.20, 95% CI: 6.29–8.24) risks for developing HE, respectively (**Table 2**). The associated risk of HE was significantly increased with levofloxacin than with cephalosporins (aOR = 5.13, 95% CI: 2.28–11.52) and penicillin (aOR = 9.40, 95% CI: 2.25–39.24) (**Tables 3 and 4**). The joint effect analysis displayed that the risk of developing HE associated with levofloxacin was increased when used in combination with insulin (aOR = 8.42, 95% CI: 1.91–37.00) or sulfonylurea (aOR = 3.56, 95% CI: 1.12–11.33) (**Table 5**).

**Table 2:** Comparison of varied antibiotics (users Vs. non-users) as exposures towards the risk of developing hypoglycemia emergency (event).

	Hypoglycemic emergency	Crude OR	Adjusted OR
	(n = 4,196)	(95% CI)	(95% CI)
**Antibiotic type (reference = non-)**			
**Cephalosporin**	2212 (52.71)	6.78 (6.42–7.16) *	6.12 (5.74–6.52) *
**Penicillin**	220 (5.24)	3.12 (2.68–3.63) *	3.10 (2.61–3.69) *
**Fluoroquinolone**	909 (21.66)	15.70 (14.15–17.42) *	12.05 (10.66–13.61) *
**Macrolide**	131 (3.12)	6.63 (5.82–7.55) *	6.85 (5.91–7.96) *
**Sulfonamide**	514 (12.25)	8.05 (7.17–9.04) *	7.20 (6.29–8.24) *
**Tetracycline**	128 (3.05)	2.28 (1.88–2.76) *	2.13 (1.71–2.64) *
**Metronidazole**	82 (1.95)	4.33 (3.34–5.61) *	3.64 (2.68–4.94) *

CI: Confidence interval; OR: Odds ratio; Adjusted OR: Adjusted for usual medication, and aDCSI score in the logistic regression model.

*P <0.05 All results were with a significant P value.

**Table 3:** Comparison of different types of quinolones and cephalosporins usage as exposure towards the risk of developing hypoglycemia emergency (event).

	Hypoglycemia emergency	Crude OR	Adjusted OR
	(n = 2,247)	(95% CI)	(95% CI)
**Antibiotic type** ^**#**^			
**Cephalosporins**	2201 (97.95)	1.00 (reference)	1.00 (reference)
**Ciprofloxacin**	10 (0.45)	2.66 (0.96–7.32)	2.64 (0.79–8.82)
**Moxifloxacin**	8 (0.35)	4.25 (1.13–16.04)*	2.87 (0.63–13.13)
**Levofloxacin**	28 (1.25)	4.06 (2.02–8.17)*	5.13 (2.28–11.52)*

CI, confidence interval; OR, odds ratio, #The cephalosporins, ciprofloxacin, moxifloxacin, and levofloxacin were independent exposures, Adjusted OR: Adjusted for usual medication, and aDCSI score in the logistic regression model.

*P <0.05.

**Table 4:** Comparison of usage of different types of quinolones and penicillins as exposures towards risk of developing hypoglycemia emergency (event).

	Hypoglycemia emergency	Crude OR	Adjusted OR
	(n = 1,106)	(95% CI)	(95% CI)
**Antibiotic type** ^**#**^			
**Penicillins**	1085	1.00(reference)	1.00(reference)
**Ciprofloxacin**	7	-	-
**Moxifloxacin**	2	-	-
**Levofloxacin**	12	4.73(1.33–16.79)*	9.40(2.25–39.24)*

CI, confidence interval; OR, odds ratio;

^#^Antibiotic drugs: penicillins, ciprofloxacin, moxifloxacin, and levofloxacin were independent exposures. Adjusted OR: Adjusted for usual medication, and aDCSI score in the logistic regression model.

*P <0.05.

**Table 5:** Joint effect analyses of oral levofloxacin and antidiabetic drugs on the risk for hypoglycemic emergency.

Levofloxacin	Biguanides	DDP-4 inhibitors	Sulfonylureas	TZDs	Insulin	Total n^#^ (levofloxacin)	Hypoglycemia, n	Adjusted OR	P value
**+**	**+**	-	-	-	-	24	10	1 (ref)	
**+**	-	**+**	-	-	-	1	0	-	-
**+**	**-**	**-**	**+**	**-**	**-**	84	60	3.56 (1.12–11.33)	0.031*
**+**	-	-	-	**+**	-	1	0	-	-
**+**	**-**	**-**	**-**	**-**	**+**	40	33	8.42 (1.91–37.00)	0.004**

Adjusted OR: Usual medication, and aDCSI score in the logistic regression model.

^#^Represent the total number of levofloxacin users with and without hypoglycemic emergency.

## Discussion

To the best of our knowledge, this is the largest case-control study that compares the association of the risk of HE associated with use of oral levofloxacin and with that of other antibiotics such as cephalosporins and penicillin. We demonstrated that compared with no antibiotic use, the intake of fluoroquinolone and sulfonamide antibiotics were associated with the highest (12.05-fold) and second (7.20-fold) highest risks for HE, respectively. Compared with cephalosporins, levofloxacin had a 5.13-fold increased risk for HE whereas when compared with penicillin antibiotics, levofloxacin had a 9.40-fold increased risk for HE. Moreover, the results identified that the risk for HE was the highest with levofloxacin, followed by moxifloxacin and ciprofloxacin. Furthermore, patients for whom levofloxacin was concomitantly prescribed with insulin or sulfonylurea were prone to develop HE.

On July 10, 2018, the FDA published a drug safety communication on the risks of developing hyperglycemia, hypoglycemia, and impairment of mental health with fluoroquinolone use [11]. Most of the supporting data of the FDA were from published case reports or series. In the cited reference articles, macrolide antibiotics were frequently set as the reference to compare fluoroquinolone antibiotics with, because of some similar indications [28,29]. In Taiwan, the cost of fluoroquinolone, for tuberculosis treatment, is not p by the NHIA and is prescribed only if the following conditions in individuals aged ≥18 years are met: 1) acute exacerbation of chronic bronchitis, 2) community acquired pneumonia, 3) complicated intraabdominal infection, and 4) severe soft tissue infections. As fluoroquinolone antibiotics, such as moxifloxacin and levofloxacin, regimen is once daily dosage, they are frequently prescribed in the outpatient or ED to increase drug adherence.

A nested case-control study on 78,433 patients from January, 2006 to November, 2007, reported an increased risk for HE with levofloxacin (aOR 1.75, 95% CI 1.12–2.93); ciprofloxacin (aOR 1.87, 95% CI 1.20–4.12); and moxifloxacin (aOR 2.48, 95% CI 1.50–4.12), compared to that with macrolides [29]. On the other hand, in the present 15-year study on a larger number of patients, a stricter criterion was adopted, where antibiotic treatment was initiated one week prior to the index date of HE, compared with one month interval of any antibiotic use in the previous study. Moreover, in this present study, to increase the precision, only the first episode of HE requiring ED visit was recorded; HE events during hospitalization were not included. Furthermore, prior studies used macrolides (such as Azythromycin) as the reference because of its little similar indications with that of fluoroquinolones. However, fluoroquinolone antibiotics are usually prescribed for indications that are different from those of macrolide antibiotics. Therefore, to control for any indication bias if any on the choice of antibiotics, PS matching was conducted in this study. Therefore, to control for any indication bias, if any, on the choice of antibiotics, PS matching was conducted in this study. In addition, cephalosporin and penicillin antibiotics were used as the reference for comparison with fluoroquinolones, because cephalosporin and penicillin antibiotics were seldom reported to induce HE. Therefore, the results reported through this study was more convincing. Another cohort study by the Veterans Affairs Healthcare System demonstrated that the adjusted OR for HE was significantly greater with levofloxacin than with azithromycin [30,31].

The mechanism of fluoroquinolone-induced hypoglycemia should be further investigated because of its widespread worldwide use. Animal studies demonstrated increased insulin secretion from rat pancreatic islet cells through blockage of the adenosine triphosphate-dependent potassium channels after exposure to fluoroquinolone antibiotics. Another reported mechanism of fluoroquinolone antibiotics, which was similar to that of sulfonylurea drugs, was enhancement of calcium influx, which can help release insulin-filled vesicles that induce HE [32–34]. Fluroquinolone antibiotics could interact with the cytochrome P450 2C9 enzyme, which is the primary pathway responsible for metabolizing glyburide, glimepiride, and glipizide. This might help explain the observation that patients with diabetes treated concomitantly with sulfonylurea were likely to develop HE.

### Strength and limitation

Till date, this is the largest cohort study that evaluates the risk associated with the use of fluoroquinolone antibiotics, especially oral levofloxacin, when treating infections in patients with T2D. PS matching was conducted to eliminate indication bias for all antibiotics rather than using macrolides as the reference, based on its some similar indications with fluoroquinolone antibiotics. The selection of case and matched control subjects was done to ensure that the matched pairs (1:10) had taken certain oral antibiotics in the matched calendar year. Even with the development of antidiabetic drugs, sulfonylureas and insulin remain popular medications to control blood sugar. In this study, joint effect analysis was conducted to compare the risk level between the combinations of levofloxacin and antidiabetic drugs. Therefore, the study results would be of much help in clinical practice. At the same time, we provided data that would strongly support the FDA warning on the side effects of hypoglycemia with fluoroquinolone use. Finally, this study included T2D severity, as represented by the aDCSI score.

One of the limitations of this study was the lack of serial laboratory data such as HbA1c and daily blood sugar records. Nevertheless, as medical resources in Taiwan are readily available, patients with diabetes are believed to receive adequate adjustment of antidiabetic drugs to achieve acceptable HbA1c levels. Next, the infection sites were not reported in Table 1 because most of the clinics and out-patient department of general or regional hospital did not provide precise disease coding to the National Health Insurance Administration. For the validation process, 100 cases who were being prescribed with oral antibiotics were randomly selected. It was surprising that most of the diagnosis codes were fever (>50%) or acute respiratory infection or acute gastroenteritis. This is why the infection sites were not included as one of the variables in for PS matching. In fact, it was impossible to conduct a perfect match, especially in the frequency-matching. Furthermore, although several important potential confounding variables is collected, it was impossible not to miss any confounders, whether measurable or non-measurable. Hence, controlling for potential confounders is not comprehensive in this study. However, PS matching was conducted on the known confounders to remove bias, as much as possible. The infection severity also remained a serious concern and a difficult dilemma. Only subjects (case and control groups) whose condition were feasible to be treated at the out-patient department (OPD) by oral antibiotics, were included for analysis. This ensured that the infection severity between the two groups (HE and no-HE) was nearly identical. Lastly, all findings from this study will be validated in the future through independent datasets.

## Conclusions

This is the largest case-control study, till date, that utilized a nationwide database with PS matching, to demonstrate that the use of oral levofloxacin, compared with other antibiotics, was significantly associated with the risk for HE in patients with T2D. Future studies on independent datasets from Taiwan and other ethnicities will be conducted to validate the findings from this study. Based on the findings from this study, clinicians should take caution in prescribing levofloxacin, especially when combined with insulin or sulfonylurea.
